# Validation of Application SuperDuplicates (AS) Enumeration Tool for Free-Roaming Dogs (FRD) in Urban Settings of Panchkula Municipal Corporation in North India

**DOI:** 10.3389/fvets.2019.00173

**Published:** 2019-06-06

**Authors:** Harish Kumar Tiwari, Ian D. Robertson, Mark O'Dea, Jully Gogoi-Tiwari, Pranav Panvalkar, Rajinder Singh Bajwa, Abi Tamim Vanak

**Affiliations:** ^1^School of Veterinary Medicine, Murdoch University, Perth, WA, Australia; ^2^Ausvet, Fremantle, WA, Australia; ^3^Ashoka Trust for Research in Ecology and the Environment, Bangalore, India; ^4^China-Australia Joint Research and Training Center for Veterinary Epidemiology, Huazhong Agricultural University, Wuhan, China; ^5^School of Pharmacy and Biomedical Sciences, Curtin Health Innovation Research Institute, Curtin University, Perth, WA, Australia; ^6^Veterinary Hospital, Union Territory of Chandigarh, Chandigarh, India; ^7^Wellcome Trust/DBT India-Alliance Fellow, Hyderabad, India; ^8^School of Life Sciences, University of KwaZulu-Natal, Durban, South Africa

**Keywords:** free-roaming dogs, application superduplicates, enumeration, rabies, dog-population, population-control

## Abstract

A cost-effective estimation of the number of free-roaming dogs is an essential prerequisite for the control of rabies in countries where the disease is endemic, as vaccination of at least 70% of the population is recommended to effectively control the disease. Although estimating the population size through sight-resight based maximum likelihood methodology generates an estimate closest to the actual size, it requires at least five survey efforts to achieve this. In a rural setting in India, a reliable estimate of at least 70% of the likely true population of free-roaming dogs was obtained with the Application SuperDuplicates shinyapp online tool using a photographic sight-resight technique through just two surveys. We tested the wider applicability of this method by validating its use in urban settings in India. Sight-resight surveys of free-roaming dogs were conducted in 15 sectors of the Panchkula Municipal Corporation in north India during September- October 2016. A total of 1,408 unique dogs were identified through 3,465 sightings on 14 survey tracks. The estimates obtained by the Application SuperDuplicates shinyapp online tool after two surveys were compared with the maximum likelihood estimates and it was found that the former, after two surveys, provided an estimate that was at least 70% of that obtained by the latter after 5–6 surveys. Thus, the Application SuperDuplicates shinyapp online tool provides an efficient means for estimating the minimum number of free-roaming dogs to vaccinate with a considerably lower effort than the traditional mark-resight based methods. We recommend use of this tool for estimating the vaccination target of free-roaming dogs prior to undertaking mass vaccination efforts against rabies.

## Introduction

Free-roaming dogs (FRD) are a serious public health problem in most urban societies of the developing world (1–3) and play an important role in the spread of dog-bite related rabies in countries where the disease is endemic (4). Local governments apply several strategies, including fertility control programmes (5), garbage management and relocation to animal shelters, to limit the size of the FRD population. However, due to infrequent and/or sporadic implementation, these methods have had little impact on the population size of FRD (6). Mass vaccination of FRD against rabies has been advocated as a practical and effective intervention to prevent dog-bite related rabies in countries where it is endemic (4, 7, 8). However, mass vaccination campaigns require 70% coverage to develop critical herd immunity against the virus in the target FRD population (6, 9, 10). Therefore, a lack of information about the true population size of FRD leads to uncertainty if a mass vaccination campaign successfully achieves the necessary level of population coverage (11).

The significance of reliably estimating the FRD population size is important to: assess the impacts of rabies control interventions, dog population management and effect on dog welfare; and to reduce the threat to wildlife (12–15). Enumeration of the FRD population, however, is a time and labor intensive exercise. An accurate estimate of the number of FRD at any given time depends on a number of factors, such as socio-economic status, cultural and social beliefs of the human population and characteristics of the habitat that influence the dynamics of the FRD population (16–18). Many enumeration methods have been employed by researchers to estimate the FRD population in different parts of the world, although the accuracy of such estimates can be questionable (15, 19). In the absence of a gold standard, population estimation methods used for wildlife that are based on the maximum likelihood estimates (MLE) using photographic sight-resight surveys have found acceptance for enumeration of FRD (20). However, these methods require multiple surveys to obtain robust estimates. A methodology that can provide a reliable estimate of the FRD population in an area using a minimum of resources is better suited to the effective implementation of a mass vaccination programme against canine rabies (21).

In an earlier study, Tiwari et al. (21) compared eight methods to enumerate FRD and found that the methods that do not take into account the individual heterogeneity of the dogs potentially underestimate the population size. The Huggin's heterogeneity models (M_h_ or M_th_) with suitable estimators (Jackknife/Chao) were shown to yield robust estimates depending upon if the surveys had reached saturation or not. The number of enumeration surveys conducted is central to the robustness of the estimates, with at least five surveys required to obtain an estimate close to the true population [(22), 69]. However, conducting surveys for 5–6 occasions is not only challenging and resource intensive but may also result in bias from surveyor fatigue (23). To overcome these challenges, an online tool based on the Good-Turing formula to assess species richness, called Application SuperDuplicates (AS), has been shown to provide a robust estimate that is equal to or >70% of the FRD population size with only two consecutive surveys (24). Tiwari et al. (21) concluded that the AS tool (https://Chao.shinyapps.io/SuperDuplicates/), a freely accessible web based calculator could be used to estimate the minimum target FRD population requiring vaccinating with minimal resource implications to control dog-bite related rabies. However, that study only assessed the AS tool in a single rural location. In this study, we test if the AS tool is as effective in urban areas where there are more complex environments and higher dog densities. Assuming that the MLE using heterogeneity models with suitable estimators provides an estimate closest to the true population size, we compare this method with the AS tool to estimate the FRD population size in the urban sectors of the Municipal Corporation of Panchkula in northern India.

## Materials and Methods

### Study Area

The study was conducted in the wards administrated by the Municipal Corporation, Panchkula in Haryana state in north India during September-October 2016. The municipal area is divided into 20 wards that are subdivided into sectors which contain residential, administrative and industrial sectors or unorganized colonies (slums) (25) (Supplementary Figures 1A,B). The selected sectors/colonies were surveyed through predetermined roads/tracks frequented by humans and FRD. A total of 15 sectors were selected from 7 wards using purposive (industrial, administrative and mixed sectors) and random (residential sectors including unorganized colonies or slums) sampling (Supplementary Figure 2). Two of the administrative sectors were surveyed together (sectors 1 and 5) resulting in a total of 14 survey tracks. Eight residential sectors (sectors 2, 7, 8, 9, 12, 16, 17, and 18) were randomly selected from the 16 available in the 7 selected wards. A mixed residential sector (sector 6) that contained public amenities including a public park, an educational institute and a hospital was also included. All the industrial sectors (IAP I and IAP II) and the unorganized colonies (Budhanpur—BP, Rajeev Colony—RC, Indira Colony—IC) in the Panchkula Municipal Corporation area were included in the study. The administrative and industrial sectors and the unorganized colonies do not have walled perimeter boundaries; however, the movement of FRD between the residential sectors is restricted by solid brick perimeter walls. To account for the presence of FRD on the roads connecting to the residential sectors, we also surveyed the surrounding roads of one of the sectors [Sector 8(P)].

### Field Methodology

The field methodology is described in detail in Tiwari et al. (21). Briefly, the selected sectors were traversed by a team of two observers riding a motorcycle at a constant speed of ~20 km/h following a predetermined route (survey-track) on alternate mornings and evenings for five or six occasions. The teams were equipped with a GPS device (Garmin eTrex20 GPS device, www.garmin.com), a digital camera and writing materials. The survey-tracks and the teams remained unchanged throughout the survey period. The FRD count survey was conducted during mornings (6–8 AM) and afternoons (4–6 PM), except for the survey-track covering the three unorganized colonies (BP, RC, and IC) where only morning counts were undertaken as photographic surveys were opposed by the residents during afternoons. The survey was conducted in each sector along the connecting bitumen roads.

For the purpose of this study we followed the definition of FRD by Beck (1) that covers both owned and unowned dog population, “Any dog observed without human supervision on public property or on private property with immediate unrestrained access to public property.” Any dog that was confined or restrained was excluded from this study.

### Data Entry and Analyses

The sighting/resighting of the FRD was recorded as a “1” and if that FRD was not sighted on a subsequent survey it was recorded as a “0” in the latter survey(s). The capture history was constructed for all FRD sighted during the survey. Leslie's test was used to test equal catchability of FRD by calculating the G-statistic from the observed and expected number sighted during the sampling period and comparing this with the critical value of a Chi-squared distribution (26). Program MARK software (www.phidot.org/software/mark/docs/book) was used to analyse the recorded capture histories that were run with Huggin's closed capture heterogeneity models (M_h_ and M_th_) using Jackknife and Chao estimators. The resighting probabilities obtained from Program MARK were used to calculate the coefficient of variation (CV) of the resighting probability across the surveys in a sector. The survey data were assumed to be saturated if no new FRD were sighted during the last survey. The capture histories of the first two consecutive surveys were used for obtaining the population estimate using the online AS shinyapp tool (https://Chao.shinyapps.io/SuperDuplicates/) (24). As the estimate of the true FRD population size was not known, the percentage population coverage for each of the survey-tracks for first two surveys was calculated on the basis of MLE (M_th_-Chao and M_h_-JK estimate for the survey tracks with and without saturation, respectively). A simple numerical comparison was made between the estimates obtained by the MLE models and the AS shinyapp online tool. Odds ratios and their 95% confidence intervals were calculated using the “odds ratio” package in R (27).

## Results

A total of 1,408 unique FRD were sighted at least once across the 14 survey-tracks in Panchkula (total of 3,465 sightings). The estimates of the FRD population in the various survey–tracks using the MLE method after 5–6 surveys ranged from 44 to 212, while after two surveys using the AS shinyapp tool the estimates were 49–342 (**Table 3**). Details of the sightings in each sector, along with the climatic conditions on the day of the survey, are presented in the Supplementary Table S1. All the assumptions for capture-recapture techniques were met, except the test of equal catchability for which the G-statistic value was greater than the critical χ^2^ value at *p* = 0.05 for k-1 degrees of freedom in 6 of the sectors included in the survey (Table 1). The odds of failing the assumption of equal catchability was significantly higher for survey tracks on which lower FRD counts were observed in the afternoon compared to the morning (OR 1.18, 95% CI 1.03–1.36, *p* = 0.02). The CV of the resighting probability ranged from 0.11 to 0.36 and the survey-tracks with CV values ≥0.27 failed to conform to the assumption of equal catchability for these surveys (Table 1). The CV between the two surveys used to estimate the population size of the FRD through the AS shinyapp tool for each survey-track is presented in Table 2.

**Table 1:** Results of Leslie's test for equal catchability of free-roaming dogs in the different sectors of Panchkula during the enumeration survey carried out during September-October 2016.

**Survey-tracks**	**G–value**	**Critical χ^**2**^-value**	**Number of surveys**	***p*-value**	Coefficient of variation (CV)^**^
8	7.08	9.49	5	0.13	0.19
2	3.69	9.49	5	0.45	0.12
12	12.55	9.49	5	0.01^∧^	0.28
IAP^*^ 1	4.08	11.07	6	0.53	0.11
IAP^*^ 2	6.43	11.07	6	0.27	0.13
BP, IC, RC#	1.36	9.49	5	0.85	0.11
9	31.35	11.07	6	0.0001^∧^	0.36
17	4.06	11.07	6	0.54	0.19
16	16.07	11.07	6	0.03^∧^	0.27
1 & 5	6.93	9.49	5	0.13	0.17
8 (P)@	1.68	11.07	6	0.9	0.13
18	15.92	9.49	5	0.0031^∧^	0.32
6	12.79	11.07	6	0.02^∧^	0.30
7	12.29	11.07	6	0.03^∧^	0.27

* *IAP, Industrial Area Part; #Budhanpur, Indira Colony, Rajeev Colony; @Sector 8 perimeter*;

∧ *Significant p-values indicate that the catchability was not equal between the sampling sessions*;

** *Coefficient of variation for the resighting probability*.

**Table 2:** The Coefficient of Variation, population coverage and corresponding difference in the estimate by Maximum Likelihood and Application SuperDuplicates shinyapp tool during the free-roaming dog enumeration surveys in 14 survey tracks in Panchkula.

**Survey–tracks**	**CV^†^**	**Number of unique dogs identified after 2 surveys**	**Population size based on maximum likelihood estimate (MLE)**	**Population size based on application superduplicates estimate (AS)**	**Difference in the estimates (AS–MLE)**
1, 5	0.17	89	212	221	9
2	0.12	97	152	186	34
6	0.3	56	142	184	42
8	0.19	77	146	190	44
8(*p*) ^*^	0.13	28	44	49	5
IAP@2	0.13	108	172	208	36
BP, RC, IC#	0.11	45	131	125	−6
7	0.27	39	89	85	−4
9	0.36	82	114	130	16
12	0.28	67	100	145	45
16	0.27	82	142	342	200
17	0.19	41	78	185	107
18	0.32	62	135	162	27
IAP@1	0.11	99	190	193	3

* *Sector 8 perimeter; @IAP, Industrial Area Part; #Budhanpur, Indira Colony, Rajeev Colony*;

† *Coefficient of variation for the resighting probability*.

A point of saturation (when no new FRD were sighted on subsequent surveys) was reached in 7 of the 14 survey tracks. The estimates of the FRD population size in the surveyed sectors by the using Huggin's heterogeneity models (M_h_ and M_th_ with estimators Jackknife and Chao, respectively) and with the online AS shinyapp tool (https://Chao.shinyapps.io/SuperDuplicates/) are presented in Table 3.

**Table 3:** Estimates of the free-roaming dog population size by Maximum Likelihood (5–6 surveys) and Application SuperDuplicates shinyapp (first 2 surveys) using sight-resight techniques in different sectors of Panchkula following enumeration surveys carried out during September-October 2016.

**Survey tracks**	**Huggin's M_h_ (JK) (5–6 surveys)**		**Huggin's M_th_ (Chao) (5–6 surveys)**		**AS shiny app tool (2 surveys)**		**Total number of unique dogs observed (2 surveys)**	**Number of singletons observed (2 surveys)**
	**(estimate ± SE)^†^**	**95% CI**	**(estimate ± SE)^†^**	**95% CI**	**(estimate ± SE)^††^**	**95% CI**		
**SURVEY TRACKS WHERE NEW FRD WERE SIGHTED DURING THE LAST SURVEY (RECOMMENDED MODEL + ESTIMATOR IS M_H_-JK)**								
1, 5	212 ± 16	188–253	198 ± 15	176–235	221 ± 36	166–313	89	66
2	152 ± 7	142–170	146 ± 8	137–168	186 ± 22	152–240	97	61
6	142 ± 16	119–182	140 ± 19	114–193	184 ± 51	99–279	56	46
8	146 ± 11	131–174	141 ± 11	127–170	190 ± 32	121–199	77	57
8 (P)^*^	44 ± 4	40–59	44 ± 4	40–58	49 ± 11	36–81	28	16
IAP@ 2	172 ± 9	160–196	164 ± 7	154–183	208 ± 24	170–267	108	68
BP, RC, IC#	131 ± 15	108–168	122 ± 19	96–173	125 ± 70	79–233	45	35
**SURVEY TRACKS WHERE NO NEW FRD WERE SIGHTED DURING THE LAST SURVEY (RECOMMENDED MODEL + ESTIMATOR IS M_TH_-CHAO)**								
7	100 ± 12	84–134	89 ± 8	79–113	85 ± 21	64–114	39	27
9	117 ± 8	107–141	114 ± 7	105–133	130 ± 12	111–161	82	41
12	106 ± 6	97–122	100 ± 7	92–122	145 ± 23	107–172	67	46
16	154 ± 13	136–188	142 ± 10	129–169	342 ± 97	149–868	82	71
17	78 ± 7	69–96	78 ± 9	68–104	185 ± 130	73–697	41	36
18	140 ± 14	120–176	135 ± 15	114–176	162 ± 29	118–238	62	47
IAP@ 1	201 ± 8	188–221	190 ± 8	180–211	193 ± 23	158–249	99	63

* *Sector 8 perimeter; @IAP, Industrial Area Part; #Budhanpur, Indira Colony, Rajeev Colony*;

† *Estimates after 5–6 surveys*;

†† *Estimate after two surveys*.

## Discussion

We estimated the population size of FRD in 15 sectors of the Municipal Corporation, Panchkula using photographic capture-recapture through MLE and multiple sight-resight surveys, and compared the estimates with those obtained after only two surveys using the online AS shinyapp tool. Since individual dogs were not physically captured but photographed from a distance without disturbing their natural behavior, any variation in the count due to behavioral attributes, such as being “trap-happy” or “trap-shy” is excluded in this study. The MLE were obtained using the Huggin's heterogeneity models, namely M_h_ and M_th_ with most appropriate estimators (Jackknife/Chao) in Program MARK after 5–6 surveys (28). However, after only two surveys the online AS shinyapp tool consistently obtained estimates of the population for all sectors that were ≥70% of the MLE estimates.

We sighted more dogs in the mornings than in the afternoons (Supplementary Table S1). This temporal variation is not unexpected because FRD appear to avoid the heavy motor traffic and enhanced human activity during the times when the afternoon sessions were undertaken (29). In contrast no temporal variation was observed in the study of Tiwari et al. (21) in the rural settings as there was no apparent change in the level of human activity or traffic between the morning and afternoon sessions.

The count-data of the FRD in this urban sight-resight survey conformed to all the requisite assumptions of the capture-recapture technique, except for the “assumption of equal catchability” in 6 of the 14 survey-tracks (Table 1). This could be due to the significant difference between counts in the morning and the afternoon sessions. The likelihood of obtaining a decreased FRD-count in the afternoon was significantly higher (OR 1.18) in the survey tracks that failed the equal catchability assumption than those where the assumption of equal catchability held true. As well as the presence of less vehicular traffic in the mornings than in the afternoons of residential sectors, residents in the urban areas were observed to feed the FRD during the morning surveys but not during the afternoon surveys. This is not surprising because during a knowledge, attitudes and practices (KAP) survey of the urban residents toward FRD conducted in parallel to this study, 72% of the respondents advised they would feed a FRD (30) with most of these (77%) fed leftover food to the FRD. Such practices would attract FRD, resulting in increased sightings during the morning surveys. However, no temporal variation in FRD sightings was observed in sectors 2, 8, and 17 (Table 1). These sectors are located near highways and active market areas, where FRD are frequently sighted, irrespective of the time of the day.

The survey-tracks in six sectors (residential−7, 9, 12, 16, 18 and mixed−6) with lower *p*-values for the Leslie's test imply unequal individual catchability during survey sessions and not surprisingly also had higher CV indicating a wider spread of the resighting probability (Table 1). These tracks have a noticeable temporal difference of FRD counts between morning and afternoon sessions (Supplementary Table S1) compared to the rural survey in Shirsuphal (21).

The key finding of this study, however, is the applicability of the online AS shinyapp tool to quickly and reliably obtain a minimum target for vaccination coverage after just two surveys (Table 3). Interestingly, the online AS shinyapp tool estimates exceeded the MLE of the Huggin's heterogeneity models (except for survey track 7; and the unorganized colonies (BP, RC, IC) for which only morning sessions were run) which is not unexpected as the temporal variation of the FRD counts was wider in Panchkula. Another factor that might influence the performance of the AS shinyapp tool is the population coverage during the two surveys used for calculating the estimate. Given that the true population size of FRD is unknown, the MLE is the closest approximation of true population size, albeit with bias. Assuming that the population size of FRD in the survey areas is fixed, the bias in the MLE should decrease as the coverage increases with successive surveys. The MLE by the M_th_ model overestimates the population size when the CV <0.4 and underestimates when the CV ≥ 0.4 (31). As none of the sight-resight data from surveyed sectors used for the Huggin's models had a CV value ≥0.4, we infer that the true population size of FRD may actually be less than the MLE (Table 3). This finding is of concern for at least two of the survey tracks, namely 16 and 17 where the AS shinyapp tool estimate exceeded the MLE estimate by a large margin (Table 3) and may lead to over-estimation of the vaccination effort required than is actually needed in the area.

Unfortunately, our investigation did not observe any pattern that could relate the relationship of the CV between counts (implying catchability of FRD), percentage of population coverage (after two surveys) and the margin of difference of MLE with the AS shinyapp tool estimate (Table 2). A plausible cause for such a large margin of difference in the estimates could be the pattern in which new animals were sighted in these survey tracks. It is therefore necessary to also examine the drivers that affect the detection probability of FRD in an area to elucidate the reasons for such large variations between sectors.

As observed by Tiwari et al. (21) in rural areas, Huggin's heterogeneity model with Jackknife estimator (M_h_-JK) potentially overestimated the population size of FRD when the count reaches saturation (no new FRD sighted in the last count). Also, the estimates obtained by the online AS shinyapp tool were always >70% of the MLE of the FRD population size from two consecutive surveys (morning and afternoon sessions). However, we found that the performance of the online AS Shinyapp tool depends on the CV of the resight probability.

We restricted the number of surveys to 5 or 6 in the urban FRD enumeration as recommended by Chao et al. (31), Otis et al. (32). We followed the recommendation for using Huggin's heterogeneity models where the sampling efforts should not be <5 for closed populations if the capture probabilities are relatively small (33). Nonetheless, half the surveyed tracks reached saturation and thus we compared the AS estimates of those sectors with estimates of model-estimator combination of M_th_-Chao.

The population estimate by the AS tool depends on the first two counts and if the number of singletons is large after two surveys, or conversely, if the number of individuals resighted on the second survey is small, the estimate tends to be larger. We conclude that the AS estimates that can be obtained through only two capture-recapture surveys provides an estimate that is >70% of the highest MLE estimate of the FRD population size. This can thus be used as a surrogate for determining the minimum number of dogs to vaccinate to ensure effective vaccination coverage against rabies. However, depending on the behavioral and ownership background of the dog population, a considerable fraction of FRD could be inaccessible to parenteral vaccination, limiting achievement of 70% coverage. A combination of parenteral and oral vaccination based on the demographic characteristics of FRD is recommended to overcome this impediment.

An adequate vaccination coverage is possible in areas where a significant proportion of dogs are owned or confined through adopting a door-to-door vaccination programme. However, in developing countries, such as India, where dog–related rabies is endemic, in addition to the potential inaccessibility of FRDs for immunization (34) and a shortage of resources (35), many vaccination campaigns fail to achieve the required 70% annual immunization coverage due to a lack of information regarding the true population size of FRD in the area (11). Based on Tiwari et al. (21) in a rural area, and this study in an urban area, we thus recommend that the web enabled freely available AS online shinyapp can be used to reliably obtain a minimum estimate of the FRD population for planning mass vaccination programmes in India and other developing countries.

## Data Availability

All datasets generated for this study are included in the manuscript and/or the Supplementary Files.

## Ethics Statement

This study involved the survey of free-roaming dogs in Panchkula Municipal Corporation administrated wards in the state of Haryana, India. The administrative approval of Panchkula Municipal Corporation was obtained for the study while the ethical approval was granted by ATREE (Ashoka Trust for Research in Ecology and the Environment) Animal Ethics committee (AAEC) vide their approval letter number AAEC/101/2016.

## Author Contributions

All authors have contributed and approve the contents of this article. HT conceived and developed the study. HT, RB, and PP collected and analyzed the data. HT, IR, AV, JG-T, and MO wrote the article, provided critical revision, and helped interpret the results and implications.

### Conflict of Interest Statement

The authors declare that the research was conducted in the absence of any commercial or financial relationships that could be construed as a potential conflict of interest.
