# Supplementary material for: Validation of Application SuperDuplicates (AS) Enumeration Tool for Free-Roaming Dogs (FRD) in Urban Settings of Panchkula Municipal Corporation in North India
Source: Front Vet Sci. 2019 Jun 6;6:173. doi: 10.3389/fvets.2019.00173 (PMC6562275; doi:10.3389/fvets.2019.00173)

Supplementary table S1. Details of number of Free-roaming dogs sighted during the sight-resight surveys carried in fourteen sectors of Panchkula Municipal Corporation administrated areas of Haryana state, India along with the meteorological data for each survey session.

| Sector | Total sightings | Unique dogs sighted | Date | †Session | Count each day | Temp (^0^C) | Humidity (%) | wind velocity (km/h) | Climate |
| --- | --- | --- | --- | --- | --- | --- | --- | --- | --- |
| 8 | 250 | 112 | 16/09/2016 | M | 53 | 26 | 85 | 4 | Clear |
|  |  |  | 18/09/2016 | E | 44 | 34 | 57 | no wind | passing clouds |
|  |  |  | 21/09/2016 | M | 58 | 28 | 88 | no wind | Sunny |
|  |  |  | 22/09/2016 | E | 37 | 29 | 67 | 4 | passing clouds |
|  |  |  | 24/09/2016 | M | 58 | 26 | 82 | 4 | Sunny |
| 2 | 313 | 127 | 6/10/2016 | M | 58 | 24 | 85 | no wind | Clear |
|  |  |  | 6/10/2016 | E | 55 | 32 | 56 | no wind | Sunny |
|  |  |  | 7/10/2016 | M | 75 | 25 | 85 | no wind | Fog |
|  |  |  | 7/10/2016 | E | 61 | 31 | 61 | no wind | Sunny |
|  |  |  | 8/10/2016 | M | 64 | 24 | 85 | no wind | Fog |
| 12 | 199 | 86 | 12/10/2016 | E | 36 | 31 | 45 | 2 | Sunny |
|  |  |  | 13/10/2016 | M | 52 | 22 | 71 | no wind | Clear |
|  |  |  | 14/10/2016 | M | 51 | 21 | 72 | no wind | clear |
|  |  |  | 14/10/2016 | E | 33 | 30 | 44 | 4 | Sunny |
|  |  |  | 15/10/2016 | E | 27 | 30 | 43 | 2 | Sunny |
| IAP 1 | 452 | 168 | 3/10/2016 | M | 64 | 28 | 88 | no wind | Fog |
|  |  |  | 3/10/2016 | E | 71 | 33 | 63 | no wind | Haze |
|  |  |  | 4/10/2016 | E | 76 | 32 | 68 | no wind | Fog |
|  |  |  | 5/10/2016 | M | 83 | 27 | 79 | no wind | Fog |
|  |  |  | 5/10/2016 | E | 85 | 32 | 66 | 4 | Scattered clouds |
|  |  |  | 6/10/2016 | M | 73 | 24 | 85 | no wind | Clear |
| IAP 2 | 451 | 144 | 8/10/2016 | M | 68 | 24 | 85 | no wind | Fog |
|  |  |  | 9/10/2016 | M | 65 | 23 | 82 | no wind | Fog |
|  |  |  | 9/10/2016 | E | 76 | 31 | 53 | no wind | Sunny |
|  |  |  | 10/10/2016 | M | 88 | 24 | 78 | no wind | Scattered clouds |
|  |  |  | 10/10/2016 | E | 68 | 31 | 53 | no wind | Clear |
|  |  |  | 11/10/2016 | M | 86 | 22 | 82 | no wind | Fog |
| BP,IC,RC | 140 | 69 | 21/09/2016 | M | 29 | 28 | 88 | no wind | Sunny |
|  |  |  | 22/09/2016 | M | 26 | 25 | 82 | 7 | Partly sunny |
|  |  |  | 23/09/2016 | M | 32 | 24 | 85 | no wind | Clear |
|  |  |  | 24/09/2016 | M | 29 | 26 | 82 | 4 | Sunny |
|  |  |  | 25/09/2016 | M | 24 | 26 | 87 | 4 | Sunny |
| 9 | 280 | 97 | 15/09/2016 | M | 65 | 26 | 84 | no wind | Sunny |
|  |  |  | 15/09/2016 | E | 30 | 32 | 63 | no wind | passing clouds |
|  |  |  | 16/09/2016 | E | 46 | 33 | 58 | 2 | sunny |
|  |  |  | 18/09/2016 | M | 58 | 27 | 85 | no wind | Fog |
|  |  |  | 20/09/2016 | M | 57 | 27 | 89 | no wind | Fog |
|  |  |  | 20/09/2016 | E | 24 | 33 | 74 | no wind | passing clouds |
| 17 | 136 | 60 | 16/09/2016 | M | 25 | 26 | 85 | 4 | Clear |
|  |  |  | 16/09/2016 | E | 21 | 33 | 58 | 2 | sunny |
|  |  |  | 17/09/2016 | M | 28 | 27 | 87 | no wind | Sunny |
|  |  |  | 17/09/2016 | E | 18 | 33 | 62 | 2 | Scattered clouds |
|  |  |  | 18/09/2016 | M | 26 | 26 | 85 | 6 | passing clouds |
|  |  |  | 18/09/2016 | E | 18 | 34 | 57 | no wind | passing clouds |
| 16 | 277 | 114 | 25/09/2016 | M | 60 | 27 | 89 | no wind | passing clouds |
|  |  |  | 25/09/2016 | E | 33 | 31 | 64 | 2 | passing clouds |
|  |  |  | 26/09/2016 | M | 54 | 26 | 87 | 4 | Fog |
|  |  |  | 27/09/2016 | M | 57 | 26 | 85 | no wind | Fog |
|  |  |  | 30/09/2016 | E | 38 | 33 | 64 | no wind | Sunny |
|  |  |  | 1/10/2016 | E | 35 | 33 | 69 | no wind | Sunny |
| 1,5 | 308 | 148 | 12/09/2016 | M | 63 | 32 | 58 | 4 | Sunny |
|  |  |  | 14/09/2016 | E | 49 | 32 | 64 | 4 | passing clouds |
|  |  |  | 15/09/2016 | E | 63 | 32 | 63 | no wind | passing clouds |
|  |  |  | 16/09/2016 | M | 77 | 26 | 88 | no wind | Clear |
|  |  |  | 16/09/2016 | E | 56 | 26 | 85 | 4 | Clear |
| 8 (P) | 120 | 37 | 8/09/2016 | M | 18 | 28 | 87 | no wind | Clear |
|  |  |  | 8/09/2016 | E | 22 | 30 | 78 | no wind | Clear |
|  |  |  | 9/09/2016 | M | 19 | 28 | 85 | no wind | passing clouds |
|  |  |  | 10/09/2016 | M | 17 | 26 | 65 | 2 | Sunny |
|  |  |  | 12/09/2016 | E | 20 | 32 | 58 | 4 | Sunny |
|  |  |  | 15/09/2016 | E | 24 | 32 | 63 | no wind | passing clouds |
| 18 | 183 | 92 | 10/09/2016 | M | 31 | 28 | 73 | 4 | passing clouds |
|  |  |  | 10/09/2016 | E | 28 | 33 | 59 | 2 | Sunny |
|  |  |  | 11/09/2016 | M | 46 | 28 | 70 | no wind | Clear |
|  |  |  | 11/09/2016 | E | 53 | 33 | 58 | 6 | passing clouds |
|  |  |  | 12/09/2016 | M | 25 | 32 | 58 | 4 | Sunny |
| 6 | 164 | 85 | 9/09/2016 | M | 26 | 28 | 85 | no wind | passing clouds |
|  |  |  | 10/09/2016 | M | 40 | 28 | 73 | 4 | passing clouds |
|  |  |  | 10/09/2016 | E | 18 | 33 | 59 | 2 | Sunny |
|  |  |  | 11/09/2016 | M | 33 | 28 | 70 | no wind | Clear |
|  |  |  | 13/09/2016 | E | 19 | 29 | 64 | 4 | Partly sunny |
|  |  |  | 14/09/2016 | M | 28 | 32 | 64 | 4 | passing clouds |
| 7 | 192 | 69 | 7/09/2016 | E | 19 | 32 | 62 | 4 | passing clouds |
|  |  |  | 8/09/2016 | M | 32 | 28 | 87 | no wind | Clear |
|  |  |  | 8/09/2016 | E | 35 | 30 | 78 | no wind | Clear |
|  |  |  | 9/09/2016 | M | 40 | 28 | 85 | no wind | passing clouds |
|  |  |  | 10/09/2016 | M | 41 | 26 | 65 | 2 | Sunny |
|  |  |  | 11/09/2016 | E | 25 | 33 | 58 | 6 | passing clouds |

†Session (E=Evening, M=Morning); ^ Temp= Temperature.

*Industrial Area Part 1, # Industrial Area Part 2, @ Budhanpur, Indira Colony, Rajeev Colony, ^ Sector 8 perimeter

Supplementary figure 1A&B. Geographical location and surveyed sectors of Panchkula that were selected for Enumeration survey during September-October 2016.


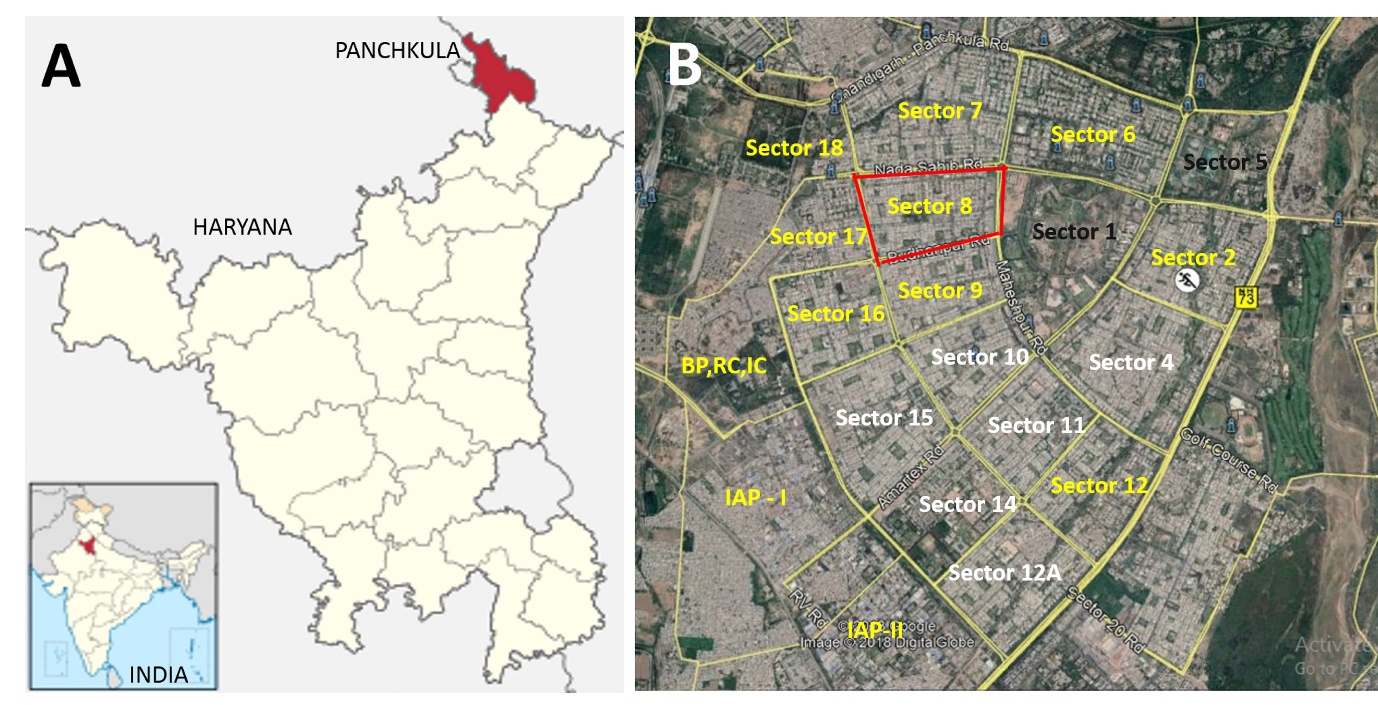


A. The geographical location of the study site relative to India and the state of Haryana.

B. Imagery of site of urban survey (*Source*: “Panchkula, Haryana state, India.” 30^0^38’58.58” N and 76^0^49’52.73” E. **Google Earth**. May 5, 2018. July 21, 2018). The sectors on which survey-tracks were traversed are in yellow and black font. Sector 1&5 were covered by a single survey-track. The red perimeter around sector 8 formed the perimeter survey-track 8(P).

Supplementary figure 2. Flow chart of the sampling strategy for selection of the survey-tracks in 15 sectors of Municipal Corporation, Panchkula for the free-roaming dog enumeration survey during September-October 2016


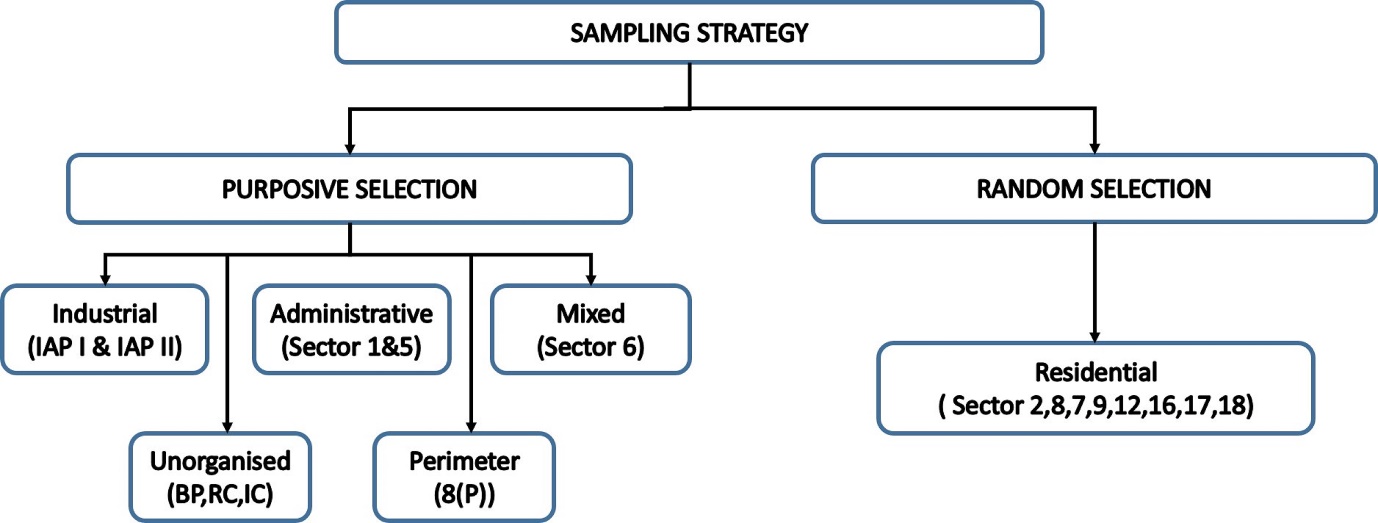

Supplement: Supplementary file 1 [file Data_Sheet_1.docx]
